# Pea (*Pisum sativum* l.) Plant Shapes Its Rhizosphere Microbiome for Nutrient Uptake and Stress Amelioration in Acidic Soils of the North-East Region of India

**DOI:** 10.3389/fmicb.2020.00968

**Published:** 2020-06-03

**Authors:** Diptaraj Chaudhari, Krishnappa Rangappa, Anup Das, Jayanta Layek, Savita Basavaraj, Basant Kumar Kandpal, Yogesh Shouche, Praveen Rahi

**Affiliations:** ^1^National Center for Microbial Resource, National Center for Cell Science, Pune, India; ^2^ICAR Research Complex for North Eastern Hill Region, Umiam, India

**Keywords:** conservation agriculture, residue management, proteobacteria, rhizosphere, tillage

## Abstract

Rhizosphere microbiome significantly influences plant growth and productivity. Legume crops such as pea have often been used as a rotation crop along with rice cultivation in long-term conservation agriculture experiments in the acidic soils of the northeast region of India. It is essential to understand how the pea plant influences the soil communities and shapes its rhizosphere microbiome. It is also expected that the long-term application of nutrients and tillage practices may also have a lasting effect on the rhizosphere and soil communities. In this study, we estimated the bacterial communities by 16S rRNA gene amplicon sequencing of pea rhizosphere and bulk soils from a long-term experiment with multiple nutrient management practices and different tillage history. We also used Tax4Fun to predict the functions of bacterial communities. Quantitative polymerase chain reaction (qPCR) was used to estimate the abundance of total bacterial and members of Firmicutes in the rhizosphere and bulk soils. The results showed that bacterial diversity was significantly higher in the rhizosphere in comparison to bulk soils. A higher abundance of Proteobacteria was recorded in the rhizosphere, whereas the bulk soils have higher proportions of Firmicutes. At the genus level, proportions of *Rhizobium*, *Pseudomonas*, *Pantoea*, *Nitrobacter*, *Enterobacter*, and *Sphingomonas* were significantly higher in the rhizosphere. At the same time, *Massilia*, *Paenibacillus*, and *Planomicrobium* were more abundant in the bulk soils. Higher abundance of genes reported for plant growth promotion and several other genes, including iron complex outer membrane receptor, cobalt-zinc-cadmium resistance, sigma-70 factor, and ribonuclease E, was predicted in the rhizosphere samples in comparison to bulk soils, indicating that the pea plants shape their rhizosphere microbiome, plausibly to meet its requirements for nutrient uptake and stress amelioration.

## Introduction

Intensive agriculture practiced to meet the food production needs of the ever-increasing population in the changing climate is posing a severe strain to soil and environmental health. Adopting climate-friendly practices appears to be the best strategy to achieve sustainability in agriculture (Rahi, 2017). Conservation agriculture (CA) is an evolved agroecosystem management approach for preserving and enriching the environmental resources while improving and sustaining the crop productivity amid possible environmental stresses (Das et al., 2014; Godfray and Garnett, 2014; Mango et al., 2017; Muzangwa et al., 2017). Comprehensively, CA requires the simultaneous application of viable crop rotations, minimal soil disturbances, and crop residue retention (Das et al., 2014, 2017; Kuotsu et al., 2014; Nichols et al., 2015). Incorporation of crop rotation and diversifying cropping systems with legume crops, including pea, lentil, and chickpea, is a key step toward CA, which increases the crop yield and decreases the impact of intensive cultivation on the environment (Laik et al., 2014; Gan et al., 2015).

The northeast region of India is ideally characterized by fragility, marginality, inaccessibility, and ecosystem diversity. Generally, the monocropping system of rice (*Oryza sativa* L.) cultivation is practiced in the northeast region of India (Das et al., 2018). Zero tillage cultivation of pea (*Pisum sativum* L.) has been considered beneficial to enhance the cropping intensity in the region (Das et al., 2018). Several strategies, including no-till, minimal tillage, and conventional tillage; *in situ* residue retention (ISRR); weed biomass (WB); green leaf manure (GLM) and farmyard manure; and rotation of peas and rice, are being tested on experimental farms of the ICAR Research Complex for North Eastern Hill Region, Umiam, Meghalaya, India (Das et al., 2014, 2017, 2018; Kuotsu et al., 2014). Although pea is cultivated under no-till and recommended dose of fertilizer and farmyard manure, it is likely that the residual effect of different tillage and nutrient application treatments followed for Kharif rice imminently influence pea growth and productivity during rabi season. The impact of reduced tillage intensity has been observed on the northeast India soils, as it potentially reverses the soil degradation and improves soil quality and crop productivity (Das et al., 2014; Kuotsu et al., 2014), but there are no reports on their impact on the soil microbial communities.

The majority of the soils in northeast regions of India are acidic (pH 5.0–6.0) and rich in organic carbon, which makes them deficient in available phosphorus, medium to low in available potassium, and highly rich in iron and aluminum (Thakuria et al., 2016). The acidic soils pose a severe challenge for pea cultivation due to the high availability of aluminum (Al), iron (Fe), and manganese (Mn), which can be toxic to the plants (Bojórquez-Quintal et al., 2017). The high availability of Fe and Al also leads to low availability of certain essential elements such as phosphorus, calcium, magnesium, and molybdenum (Vyas et al., 2007; Dambrine, 2018). Moreover, pea plants have high phosphorus requirements for nodule formation and its function and optimum photosynthesis (Powers and Thavarajah, 2019).

Microbial communities are vital indicators of soil quality and are highly sensitive to soil management practices (Fierer et al., 2007; Sánchez-Cañizares et al., 2017). Previous studies have suggested that no-till alters microbial diversity and activity significantly when compared to conventional tillage (Degrune et al., 2017; Babin et al., 2019). Similarly, nutrient and residue management practices such as the application of chemical fertilizers often influence the endogenous microbial communities (Fierer et al., 2007). However, soil bacterial diversity is known to be affected by the numerous environmental factors including soil pH (Lauber et al., 2009; Cho et al., 2016; Sánchez-Cañizares et al., 2017), the chemistry of soil organic matter (Lupatini et al., 2017), plant species grown in the soil (Yamamoto et al., 2018), secretion of different root exudates by the plant species (Sasse et al., 2018; Huang et al., 2019), and processing of the soil before the cultivation of the plants (Moreno-Espíndola et al., 2018). Plant root exudates primarily stimulate the microbial activity in the rhizosphere, resulting in increased microbial active biomass and abundance in the rhizosphere by several folds in comparison to surrounding bulk soil (Lugtenberg and Kamilova, 2009). During this process of increased microbial activity in plant rhizosphere, the selection of some specific microorganisms has been observed, leading to the buildup of plant-specific community in the rhizosphere (Hu et al., 2018). A recent study suggested that enrichment of members of bacterial families known for P-solubilizing, such as Rhizobiacea, Enterobacteriaceae, Pseudomonadaceae, and Burkholderiaceae, has been observed in the pea rhizosphere (Harkes et al., 2020). In comparison to cereals, legumes pose a much stronger influence on the selection of rhizosphere microbiome (Hamel et al., 2018; Harkes et al., 2019, 2020).

Conservation agriculture and the introduction of pea as a rotation crop in the acidic soils of the North East Indian region have been proven profitable and advantageous to the farmers (Das et al., 2018). Therefore, it is important to decipher the structure of microbial communities under long-term CA to develop future strategies to improve the soil health in the region and expand pea cultivation. We hypothesize that different treatments of CA will influence the microbial communities in the acidic soils of North East India. We also hypothesize that the rotation crop plant (i.e., pea) shapes its rhizosphere microbiome to meet its nutrient uptake and stress tolerance requirements in the acidic and iron-rich soils of the North East Indian region.

## Materials and Methods

### Sample Collection

Different tillage and residue management treatments are maintained for the last 8 years by alternatively cultivating rice followed by pea cultivation in the experimental fields of the ICAR Research Complex for NEH Region (950 m above mean sea level, 25°30′N latitude and 91°51′E longitude), located in Eastern Himalayan Region, India. The experimental site is characterized by a subtropical climate with mild winter and warm summer conditions. The average annual rainfall received is 2,000 mm. The maximum temperatures (25–32.3°C) were observed during July, and lower temperatures of 3°C to 14°C were recorded during the January–February months. Three different tillage treatments, that is, no-till, minimum tillage, and conventional tillage, were practiced during the rice cultivation (Das et al., 2014). Six treatments of nutrient management including 100% NPK, 50% NPK, and 50% NPK with ISRR at 5 Mg ha^–1^ (used after chopping into 10 cm pieces); 50% NPK with WB, that is, *Ambrosia artemisiifolia* (locally available weed) at 10 Mg ha^–1^ on fresh weight basis used after chopping into ∼10 cm size; 50% NPK with GLM, that is, *Tephrosia purpurea* (leguminous hedge plant grown in the fences and bunds) used at 10 Mg ha^–1^ on fresh weight basis after chopping into ∼10 cm size; and 100% organic treatment, that is, farmyard manure (at 5 t/ha) with rock phosphate (at 150 kg/ha were maintained during rice cultivation. Cultivation of pea was undertaken as a rotation crop by following a no-till practice. Pea seeds were sown at a rate of ∼80 kg/ha by opening narrow troughs of optimum depth with the help of manually operated furrow opener in between two rice lines, thus giving a row-to-row spacing of 20 cm for pea. The recommended dose of nutrients and seeds were placed in the furrow and covered with soil: Farm Yard Manure (FYM) mixture (2:1 ratio) for better seed and soil contact. As a whole, only one treatment was maintained as 100% organic treatment. In contrast, the remaining treatments were replaced with 50% inorganic treatment (20:60:40 N:P_2_O_5_: K_2_O kg/ha), along with 50% of crop residues (WB, rice ISRR, GLM) incorporation. The N, P, and K for rice and pea were supplied through urea (46% N), single superphosphate (16% P_2_O_5_), and muriate of potash (60% K_2_O), respectively. For microbial community analysis, bulk soil and pea rhizosphere samples were collected from each treatment plot. Bulk soil samples were collected in triplicates and pooled together from all the treatment plots. Three pea plants growing in the midrows of each plot were uprooted using a shovel. The whole root systems of the plants were separated from the loosely adhered soil by gentle shaking. The samples from each plot were pooled together in a sterilized polythene bag and transported to the laboratory under 4°C. All the samples were immediately processed for community DNA extraction.

### Chemical Properties of Soil Samples

Three soil samples were obtained after harvest of pea from 0 to 15 cm soil depths from each plot using a soil auger and composited. Soil samples were air dried, grinded, and passed through 2 mm sieve and used for analyzing soil fertility parameters, such as soil pH determined in 1:2 soil water suspensions with the help of combined glass electrode on microprocessor-based pH meter (Jackson, 1973). Available N was estimated by the alkaline permanganate method (Subbiah and Asija, 1956), available P by Bray’s extraction method (Bray and Kurtz, 1945) using spectrophotometer, and available K by neutral normal NH_4_OAC extraction (Knudsen et al., 1982) using flame photometer. Soil organic carbon (SOC) concentration was determined by the Walkley and Black method (Nelson and Sommers, 2005). The total carbon was determined by the dry combustion method (Nelson and Sommers, 2005) using a Total Organic Carbon (TOC) analyzer (Elementer Vario Select, Langenselbold, Germany). The SOC was assumed to be equal to the total C with negligible inorganic C concentration as the soil pH was below 7 (Jagadamma and Lal, 2010).

Soil microbial biomass carbon (SMBC) was estimated by soil fumigation technique (Anderson and Ingram, 1993; Tabatabai, 1994). Soil microbial biomass nitrogen (SMBN) was estimated by soil fumigation method (Jimenez and Ladha, 1993). Soil biomass P is calculated from the difference between the amount of inorganic P (Pi) extracted by 0.5 (Spm) NaHCO_3_ (pH 8.5) from fresh soil fumigated with CHCl_3_ and the amount extracted from nonfumigated soil (Brookes et al., 1981). Soil dehydrogenase activity (DHA) was determined by the triphenyl formazan reduction method (Casida et al., 1964).

### Isolation of Rhizosphere Soil, DNA Extraction, and Next-Generation Sequencing

Roots of pea plants were transferred to the 15 mL sterilized centrifuge tube and submerged in the 10 mL phosphate-buffered saline (PBS) to harvest rhizosphere soils. The tubes were subjected to sonication for 60 s, and roots were transferred to a fresh centrifuge tube filled with 10 mL PBS. The sonication step was repeated once again to remove the soil adhered to the roots. The process of washing in PBS and sonication was repeated three times to get the total rhizosphere soil into the PBS. The rhizosphere soil containing PBS was centrifuged at 5,000 × *g* for 10 min. The soil pellet was used for the extraction of rhizosphere metagenomic DNA. Total community DNA was extracted from bulk soil and rhizosphere soil samples using the DNeasy PowerSoil kit (Qiagen, Hilden, Germany) following the manufacturer’s instruction. The concentration of resulting DNA was measured using NanoDrop-1000 (Thermo Fisher Scientific, Waltham, United States), and DNA concentration was normalized to 10 ng/μL. The freshly extracted DNA was used as the template for the amplification of the V4 region of the bacterial 16S rRNA gene using universal bacterial primers (Fadrosh et al., 2014). The amplicon sequencing (library preparation and sequencing) was performed on the Illumina Miseq platform according to the manufacturer’s instructions (Illumina, Hayward, United States).

### Bioinformatics and Statistical Analysis

Assembly of forward and reverse reads generated for each sample was carried out using FLASH (Fast Length Adjustment of SHort reads) (Magoc and Salzberg, 2011). Bacterial diversity analysis was done using a standard QIIME (v1.9.0) pipeline (Zhernakova et al., 2016) on the assembled sequences. These sequence reads were clustered into operational taxonomic units (OTUs) using UCLUST algorithm (Edgar, 2010) and SILVA database (Quast et al., 2012) by closed reference-based OTU picking method keeping 97% sequence similarity threshold. Representative sequences (repset) from each OTU were selected for taxonomic assignment. Statistical analysis of the alpha diversity across different groups was done using STAMP (Parks and Beiko, 2010). The differential abundance analysis of bacterial genera across the different study groups was also done using STAMP. The abundance of bacterial phylum and genus was represented using GraphPad Prism (GraphPad Software, La Jolla, CA, United States). Beta-diversity analysis of the bacterial diversity present in the bulk and rhizosphere soil samples was done using the online tool microbiome analyst (Dhariwal et al., 2017). R language-based package ggtern, an extension of package ggplot2, was used to plot ternary diagrams of the differential abundant OTUs in the bulk soil and rhizosphere soil samples. The presence of the shared and unique bacterial genera across the bulk soil and rhizosphere samples was investigated using the online tool InteractiVenn^1^. Operational taxonomic units–based predictive functional analysis of the bacterial community was done using the Tax4Fun package (Aßhauer et al., 2015) in R and KEGG database. Also, principal component analysis (PCA) based on the abundant predicted functions was performed in the PAST3 (PAleontological STatistics) software (Hammer et al., 2001).

### Absolute Quantification of Bulk Soil and Pea Rhizosphere Bacteria

Quantitation of total bacteria and phylum Firmicutes was done using respective qPCR primers Supplementary Table S1). Briefly, for each gene, 10 μL reactions (in triplicate) were set containing suitable pairs of primers (0.5 μM), 10 ng of metagenomic DNA and SYBR green master mix (Applied Biosystems Inc., Foster City, California, United States). The reactions were run on 7300 Real-time PCR system (Applied Biosystems Inc.) using the following PCR conditions: initial denaturation at 95°C for 10 min, followed by 40 cycles at 95°C for 10 s and 60°C for 1 min. Group-specific standard curves were generated from serial dilutions of a known concentration of individual PCR products. The amplification specificity of primers was checked by melt curve analysis performed at the end of qPCR cycles. The mean values of the three replicate were used for enumerations of tested gene copy numbers for each sample using standard curves.

### Data Availability

The sequence data are made available at NCBI SRA submission with accession number SUB5624752 (Bioproject ID: PRJNA544901).

## Results

### Chemical Properties of Soil

Soil pH (1:2.5) was found to be influenced by tillage and nutrient management practices at depth 0–15 cm. Application of 50% NPK +ISRR at 5 t/ha recorded slightly higher soil pH compared to 100% NPK. It was recorded that SOC and TOC at 0–15 cm soil depth were significantly varied by tillage and nutrient management practices (Table 1). Among the nutrient management practices, the higher SOC and TOC at 0–15 cm depth of soil were recorded under 50% NPK + WB, and it was at par with 50% NPK + GLM and FYM + WB + RP, whereas lower SOC and TOC contents at 0–15 cm depth of soil were recorded under 50% NPK and 100% NPK (no residue application). The interaction effect of tillage and nutrient management practices on SOC and TOC appeared significant. Among the nutrient management practices, SOC was observed to be the highest under MT with 50% NPK + GLM (2.89%) followed by NT with 50% NPK + WB (2.83%). The lowest SOC at 0–15 cm was recorded under CT + 50% NPK (2.03%). TOC at 0–15 cm of the soil was higher under MT for FYM+WB+RP (3.25%) followed by NT + 50% NPK + GLM (3.24%) (Table 1).

**TABLE 1:** Chemical properties of soils collected from different treatment plots.

Tillage	Soil pH				SOC (%)				TOC (%)			
Nutrient Inputs	ZT	MT	CT	Mean	ZT	MT	CT	Mean	ZT	MT	CT	Mean
100% NPK	4.58	4.58	4.65	**4.60**	2.66	2.47	2.28	**2.47**	3.16	3.13	2.95	**3.08**
50% NPK	4.55	4.49	4.58	**4.54**	2.41	2.34	2.03	**2.26**	3.12	3.06	2.66	**2.95**
50% NPK+ISRR	4.84	4.76	4.62	**4.74**	2.76	2.52	2.34	**2.54**	3.25	3.20	3.19	**3.21**
50% NPK+WB	4.73	4.71	4.70	**4.71**	2.83	2.50	2.42	**2.58**	3.21	3.18	3.18	**3.19**
50% NPK+GLM	4.66	4.61	4.75	**4.67**	2.82	2.89	2.72	**2.81**	3.24	3.23	3.16	**3.21**
FYM+WB+RP	4.72	4.71	4.62	**4.68**	2.80	2.78	2.67	**2.75**	3.17	3.25	2.91	**3.11**

**Mean**	**4.68**	**4.64**	**4.65**		**2.71**	**2.58**	**2.41**		**3.19**	**3.18**	**3.01**	

**Variant**	**S.Em ±**		**C.D. (p = 0.05)**		**S.Em ±**		**C.D. (p = 0.05)**		**S.Em ±**		**C.D. (p = 0.05)**	

Tillage	0.03		0.07		0.02		0.07		0.02		0.05	
Nutrient inputs	0.04		0.05		0.03		0.05		0.03		0.04	
Interactions	0.06		0.18		0.06		0.16		0.05		0.13	

	**N (Kg/ha)**				**P (Kg/ha)**				**K (Kg/ha)**			
	**ZT**	**MT**	**CT**	**Mean**	**ZT**	**MT**	**CT**	**Mean**	**ZT**	**MT**	**CT**	**Mean**

100% NPK	270.9	271.4	264.3	**268.8**	10.6	9.9	9.1	**9.9**	207.3	201.3	187.9	**198.8**
50% NPK	261.1	252.3	244.9	**252.8**	10.4	9.1	8.7	**9.4**	197.1	190.8	183.9	**190.6**
50% NPK+ISRR	307.2	291.2	281.6	**293.3**	11.5	10.8	10.4	**10.9**	235.2	225.5	208.9	**223.2**
50% NPK+WB	312.3	306.7	298.3	**305.8**	11.3	10.9	10.3	**10.8**	219.9	218.2	199.9	**212.7**
50% NPK+GLM	308.2	307.0	298.3	**304.5**	11.2	10.5	9.8	**10.5**	218.5	209.4	196.6	**208.2**
FYM+WB+RP	286.0	297.7	276.7	**286.8**	11.1	10.6	10.5	**10.7**	234.0	213.0	214.7	**220.6**

**Mean**	**291.0**	**287.7**	**277.3**		**11.0**	**10.3**	**9.8**		**218.7**	**209.7**	**198.6**	

**Variant**	**S.Em ±**		**C.D. (*p* = 0.05)**		**S.Em ±**		**C.D. (*p* = 0.05)**		**S.Em ±**		**C.D. (*p* = 0.05)**	

Tillage	1.4		4.1		0.1		0.3		3.9		11.3	
Nutrient inputs	2.0		2.9		0.2		0.2		5.6		7.9	
Interactions	3.5		10.0		0.3		0.7		9.7		27.7	

*SOC, Soil Organic Carbon; TOC, Total Organic Carbon; N, Nitrogen; P, Phosphorus, K, Potassium; FYM, Farm Yard Manure; ISRR, in situ Rice Residue Retention; WB, Weed Biomass; GLM, Green Leaf manure; RP, Rock phosphate. Values in bold are the mean.*

The interaction effect of tillage and nutrient management practices on available N, P, and K content were significant (Table 1). Available N, P, and K contents were significantly higher under NT compared to MT and CT at both depths. Among the nutrient management practices, N recorded significantly higher under 50% NPK + GLM followed by 50% NPK + WB and 50% NPK + ISRR compared to 50% NPK, whereas available P recorded higher under 50% NPK + ISRR (11.5 kg/ha) at 0–15 cm. Available K at 0–15 cm of the soil was the higher under NT + 50% NPK + ISRR (235.2 kg/ha) followed by MT for 50% NPK + ISRR (225.5 kg/ha) (Table 1). Detailed results of other chemical properties of soils are provided in Supplementary Information.

### Diversity Analysis of Bacterial Communities

A total of 2,686,925 raw sequence reads were generated for the bulk soil (*n* = 18) and rhizosphere soil (*n* = 18) samples using the Illumina Miseq sequencing platform. Among these sequences, 2,650,426 sequences (98.6%) were assembled using FLASH and clustered into 15,048 OTUs. Among these, 10,078 OTUs were represented by two or more than two sequences, whereas remaining OTUs were singletons.

Alpha diversity assessed by observed OTU, Shannon, Simpson, and Chao1 indices differed significantly between rhizosphere and bulk soils (*p* > 0.05) (Supplementary Figure S1). The values of diversity indices were always significantly higher in rhizosphere samples in comparison to bulk soils (Supplementary Figure S1). Tillage and residue management practices do not influence any of the alpha-diversity indices (Supplementary Figure S1). Both bulk soil and rhizosphere soil samples from the organic treatment exhibited the least intersample variation in Shannon and Simpson alpha-diversity indices in comparison to other residue management treatments (Supplementary Figures S1H,K).

**FIGURE 1:**
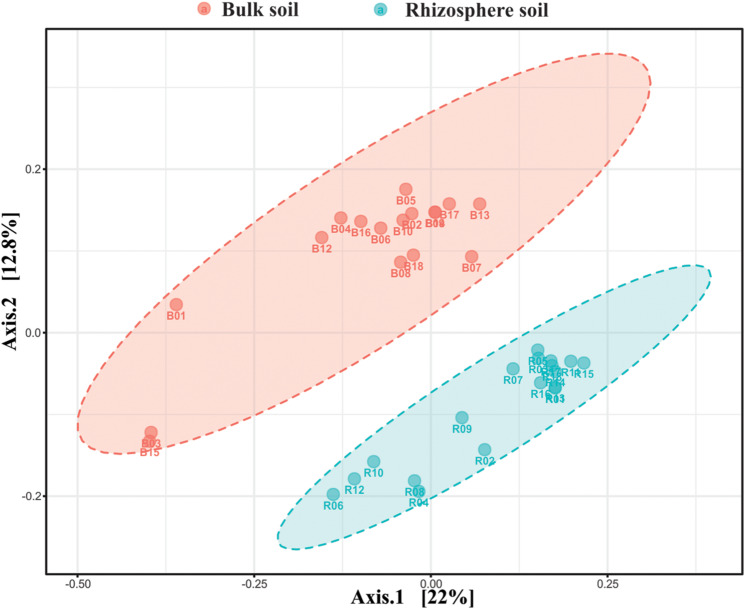
PCoA biplot based on relative abundance of bacterial OTUs exhibiting the beta diversity among the bulk soil and pea rhizosphere samples.

The beta-diversity analysis based unweighted unifrac Principal Coordinates Analysis (PCoA) showed clustering of the bulk soil and rhizosphere soil sample into two distinct clusters (Figure 1). The total variation explained by the first was PCoA 34.8% (22% for axis 1 and 12.8% for axis 2), wherein all the bulk soil samples were on the positive side of axis 2 except two samples (B15 and B03), whereas all the rhizosphere soil samples were on the negative side of axis 2.

### Taxonomic Composition of Bacterial Communities

**FIGURE 2:**
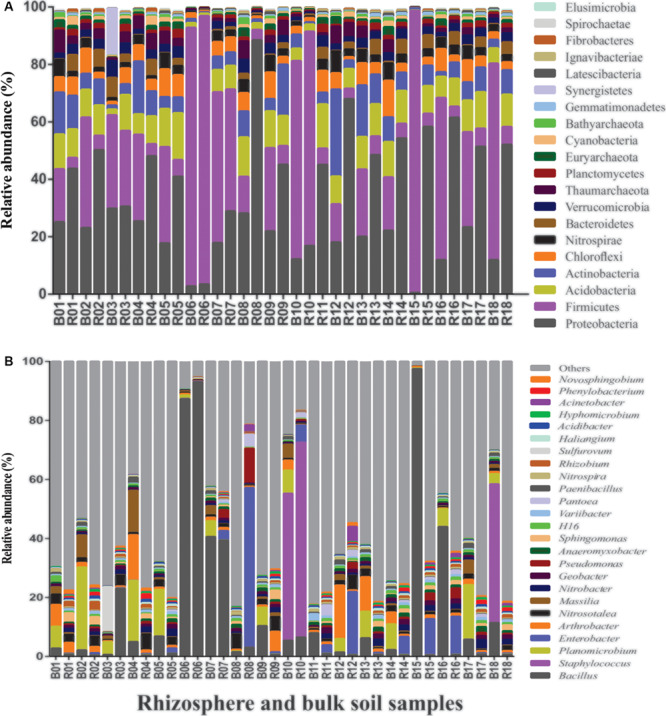
Relative abundance of bacteria in the bulk soil and pea rhizosphere samples at the phylum **(A)** and genus **(B)** levels.

A total of 71 bacterial phyla were detected in the bulk soil and rhizosphere samples. The highly abundant phyla include Proteobacteria (32.5%), Firmicutes (29.4%), Acidobacteria (9.03%), Actinobacteria (7.2%), Chloroflexi (4.7%), Nitrospirae (3.1%), Verrucomicrobia (2.7%), Thaumarchaeota (2.6%), Bacteroidetes (2.1%), and Planctomycetes (1.0%) and constituted 95% of the overall bacterial community (Figure 2A). A higher abundance of Firmicutes was recorded in bulk soil (∼41.7%) in comparison to the rhizosphere (∼17.8%). On the contrary, Proteobacteria were highly abundant in the rhizosphere (∼43.9%) in comparison to bulk soil (∼18.6%) samples (Figure 2A). At genera level, 24 bacterial genera, including *Bacillus*, *Nitrobacter*, *Pseudomonas*, *Paenibacillus*, and *Rhizobium*, were found dominant with a collective abundance of more than 0.5% (Figure 2B).

**FIGURE 3:**
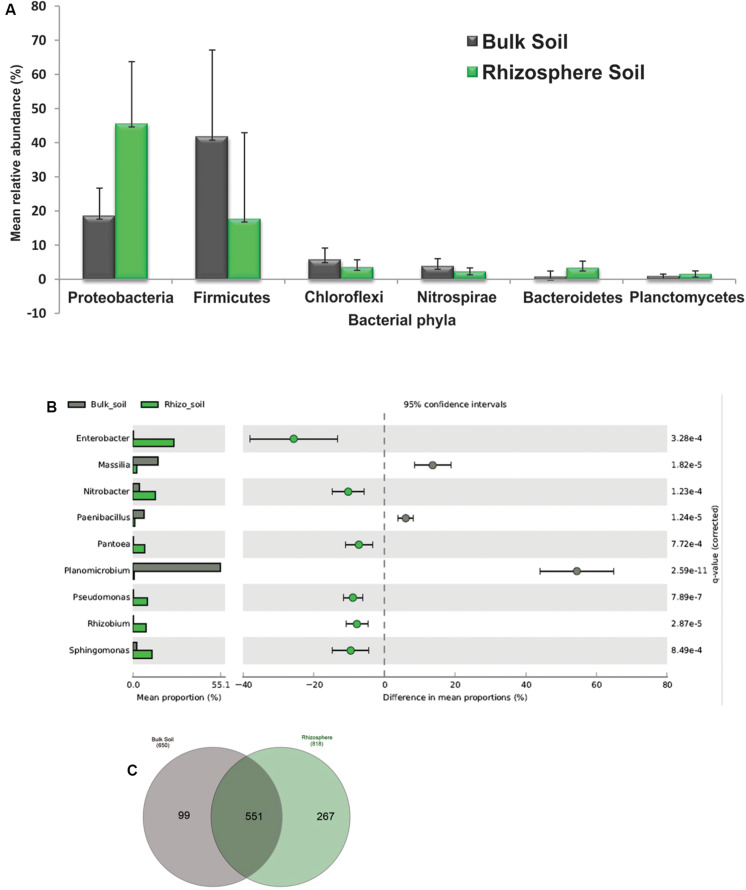
Differentially abundant bacterial taxa across the bulk soil and pea rhizosphere samples. **(A)** At the phylum level. **(B)** At the genera level. **(C)** Venn diagram representing the number of shared and unique OTUs in the bulk soil and rhizosphere soil samples.

Comparative analysis at phylum and genus taxonomic ranks revealed the differences in the abundance of bacterial taxa in bulk soil and rhizosphere soil samples (Figure 3). Significantly higher abundance of Proteobacteria and Bacteroidetes was observed in pea rhizosphere samples in comparison to bulk soil (Figure 3A). The abundance of Firmicutes, Chloroflexi, Nitrospirae, and Planctomycetes was significantly higher in bulk soil samples over rhizosphere samples (Figure 3A). The abundance of genera *Rhizobium*, *Pseudomonas*, *Pantoea*, *Paenibacillus*, *Nitrobacter*, *Enterobacter*, and *Sphingomonas* was significantly higher in rhizosphere soils, whereas *Massilia*, *Paenibacillus*, and *Planomicrobium* were highly abundant in bulk soils (*t*-test, *P* < 0.05) (Figure 3B). Among the total of 917 bacterial genera reported in this study, 551 genera (60%) were common for bulk soil and rhizosphere soil samples (Figure 3C). Rhizosphere samples showed a selection of 267 unique genera, whereas only 99 genera were exclusive to bulk soil samples.

### Quantitative Analysis of Bacterial Community

**FIGURE 4:**
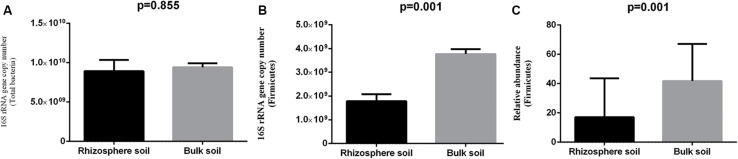
Abundance of total bacteria and phylum Firmicutes in the bulk soil and rhizosphere soil samples. **(A)** Total bacterial abundance in the bulk soil and rhizosphere soil samples, **(B)** qPCR-based and **(C)** NGS-based relative abundance of phylum Firmicutes in the bulk soil and rhizosphere soil samples.

The total bacterial abundance estimated by qPCR was statistically not different for the rhizosphere (8.9 × 10^9^ copies/gm) and bulk soil (9.42 × 10^9^ copies/gm) samples (Figure 4A). The abundance of phylum Firmicutes was significantly higher (3.76 × 10^9^ copies/gm) in the bulk soil samples in comparison to the rhizosphere samples (1.78 × 10^9^ copies/gm) (Figure 4B). The mean relative abundance of Firmicutes detected by 16S rRNA gene amplicon sequencing data was ∼18% in the rhizosphere samples and ∼42% in the bulk soil samples (Figure 4C).

### Effect of Soil Management Practices on the Bulk Soil and Pea Rhizosphere Microbiome

**FIGURE 5:**
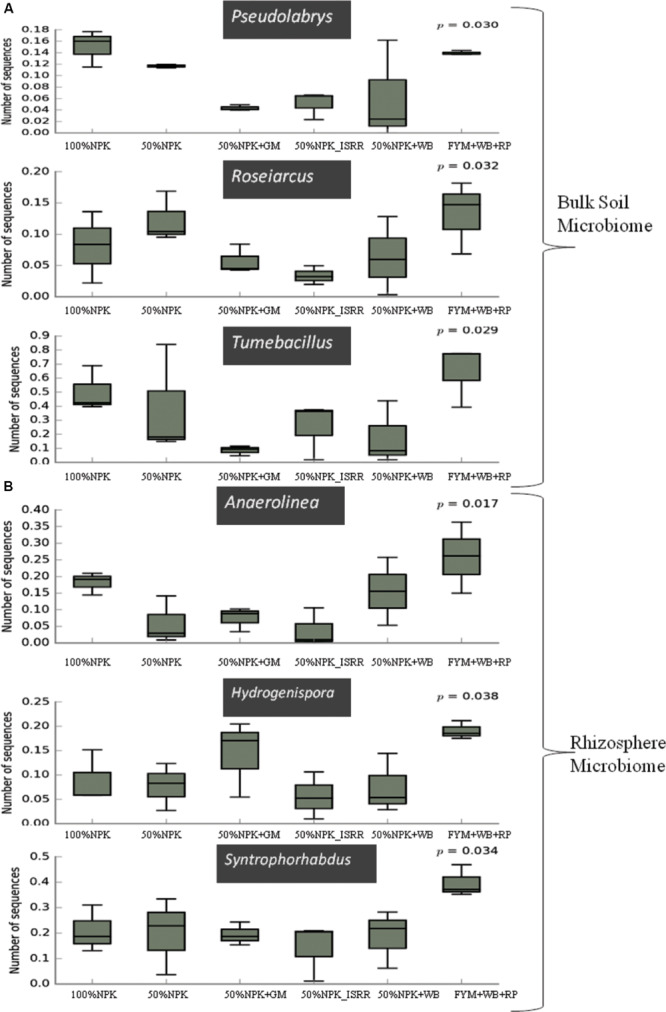
Differentially abundant bacterial taxa detected across the different residue management practice groups in the **(A)** bulk soil and **(B)** pea rhizosphere samples.

Bulk soil microbial community showed significant differences in the abundance of *Pseudolabrys*, *Roseiarcus*, and *Tumebacillus* across the residue management practices (Figure 5A). *Pseudolabrys* and *Tumebacillus* were highly abundant in samples with 100% NPK, 50% NPK, and organic treatments in comparison to remaining treatments. The abundance of *Roseiarcus* was higher in 50% NPK and organic treatment samples (Figure 5A). Significantly higher abundance of bacterial genera *Anaerolinea*, *Hydrogenispora*, and *Syntrophorhabdus* was recorded for the organic treatment among the rhizosphere samples (Figure 5B).

The impact of tillage history was also observed on the enrichment of specific OTUs in the bulk soil and rhizospheric soil (Supplementary Figure S2). A total of 66 OTUs belonging to 32 taxa were enriched in bulk soils with a history of minimum tillage. Specific enrichment of 12 OTUs belonging to four taxa was recorded for samples with conventional tillage, and only six OTUs belonging to four taxa were enriched in samples with zero tillage in bulk soils (Supplementary Figure S2A). Similarly, among the rhizosphere soil samples, significant enrichment of 295 OTUs of 96 taxa, 17 OTUs of the 13 taxa, and 82 OTUs of the 37 taxa was observed in the samples with conventional tillage, minimum tillage, and zero tillage history, respectively (Supplementary Figure S2B). Differences in the abundance of 11 genera were recorded in the rhizosphere sample across the history of different tillage treatment (Table 2). All these genera showed higher abundance in the conventional tillage fields.

**TABLE 2:** Differentially abundant bacterial genera across the three different tillage treatments practices.

Genera	No-till (Mean ± SD)	Conventional tillage (Mean ± SD)	Minimum tillage (Mean ± SD)	*p*-values (ANOVA)
*Flavobacterium*	0.989 ± 0.684	2.197 ± 0.428	0.523 ± 0.54	0.001
*Lysobacter*	0.639 ± 0.426	1.194 ± 0.354	0.269 ± 0.174	0.002
*Pseudolabrys*	1.071 ± 0.533	1.298 ± 0.158	0.345 ± 0.36	0.003
*Rhodomicrobium*	0.437 ± 0.253	0.888 ± 0.18	0.299 ± 0.264	0.003
*Pedomicrobium*	0.297 ± 0.164	0.578 ± 0.197	0.194 ± 0.15	0.008
*Acidothermus*	0.421 ± 0.233	0.836 ± 0.418	0.2 ± 0.127	0.01
*Bryobacter*	1.285 ± 0.663	1.54 ± 0.32	0.473 ± 0.463	0.011
*Anaeromyxobacter*	1.763 ± 1.184	3.445 ± 1.398	1.107 ± 0.885	0.018
*Rhizomicrobium*	0.72 ± 0.645	1.179 ± 0.423	0.274 ± 0.224	0.025
*Nitrobacter*	4.096 ± 2.36	5.912 ± 1.874	2.104 ± 1.812	0.031
*Sideroxydans*	0.401 ± 0.366	0.629 ± 0.121	0.195 ± 0.196	0.047

### Correlations Between Soil Properties and Microbial Community Structure

Significant correlations were observed between the relative abundance of a few bacterial phyla and genera in both bulk and rhizosphere samples and soil properties (Table 3 and Supplementary Table S4). However, the number of significant correlations was low in rhizosphere samples, in comparison to bulk soil samples. The relative abundance of Planctomycetes in bulk was a significantly negative correlation with six soil properties, including N, P, TOC, Ca, DHA, and SMBP (Table 3). In addition, a significant negative correlation was also observed between Chloroflexi and N, TOC, and Ca; Acidobacteria and N and DHA; Proteobacteria and Fe and Mn; and Actinobacteria and Fe, whereas Firmicutes showed a positive correlation with Mn in bulk soil. At the genera level, a significant negative correlation was recorded for the relative abundance of *Geobacter* and *Nitrobacter* in bulk soil with the majority of the soil properties, such as N, TOC, SOC, Ca, SMBC, SMBN, and SMBP; in case of *Geobacter*, pH values and P were also negatively correlated. Significant negative correlations were also observed for the relative abundance of *Nitrosotalea* in bulk soil with pH, N, Ca, SMBC, and SMBP. However, the relative abundance *Bacillus* in bulk soil was positively correlated with Fe, whereas a negative correlation between Fe and the relative abundance *Arthrobacter* and *Massilia* was remarkably evident.

**TABLE 3:** Pearson’s correlation coefficient (R) between the relative abundance of bulk soil bacterial taxa and measured soil characteristics.

Bacterial taxa	Measured soil characteristics																
	pH	N	P	K	TOC	SOC	Fe	Ca	Cu	DHA	Mg	Mn	S	SMBC	SMBN	SMBP	Zn
**Phylum**																	
Proteobacteria	–0.46	–0.32	–0.12	–0.14	–0.25	–0.02	−0.49*	–0.25	–0.18	–0.14	–0.03	−0.49*	0.02	–0.24	–0.14	–0.28	–0.24
Firmicutes	0.35	0.45	0.27	0.15	0.39	0.20	0.48	0.39	0.28	0.35	0.12	0.49*	0.07	0.31	0.24	0.37	0.27
Acidobacteria	–0.42	−0.59*	–0.4	–0.22	–0.45	–0.3	–0.3	–0.6	–0.27	−0.5*	–0.3	–0.24	–0.1	–0.33	–0.26	–0.47	–0.02
Actinobacteria	–0.05	–0.2	–0.1	0.01	–0.11	–0.15	−0.53*	–0.18	–0.4	–0.3	–0.03	–0.43	–0.07	–0.11	–0.15	–0.15	–0.42
Chloroflexi	–0.28	−0.56*	–0.47	–0.3	−0.65*	–0.4	–0.27	−0.52*	–0.32	–0.41	–0.25	–0.24	–0.2	–0.39	–0.36	–0.46	–0.05
Nitrospirae	–0.18	–0.42	–0.28	–0.07	–0.36	–0.24	–0.34	–0.44	–0.31	–0.47	–0.2	–0.26	–0.03	–0.16	–0.17	–0.3	0.01
Verrucomicrobia	–0.26	–0.21	–0.17	–0.12	–0.21	–0.07	–0.14	–0.23	–0.04	–0.21	–0.04	–0.29	0.05	–0.2	–0.07	–0.23	–0.06
Thaumarchaeota	–0.32	–0.46	–0.22	–0.09	–0.23	–0.17	–0.39	–0.38	–0.15	–0.29	–0.15	–0.4	–0.1	–0.26	–0.21	–0.33	–0.31
Bacteroidetes	0.03	0.17	0.14	0.07	0.07	0.16	0.08	0.25	0.27	0.23	0.21	–0.22	0.06	–0.03	0.08	0.10	–0.19
Planctomycetes	–0.45	−0.59*	−0.5*	–0.35	−0.61*	–0.44	–0.23	−0.62*	–0.36	−0.57*	–0.38	–0.09	–0.24	–0.41	–0.39	−0.53*	0.05
**Genera**																	
*Bacillus*	0.45	0.31	0.14	0.09	0.24	0.04	0.66*	0.21	0.37	0.21	0.02	0.43	–0.05	0.12	0.09	0.22	0.40
*Staphylococcus*	–0.18	0.16	0.04	0.00	0.21	0.09	–0.21	0.23	–0.18	0.06	–0.01	0.28	–0.02	0.21	0.09	0.14	–0.19
*Planomicrobium*	–0.27	–0.2	0.13	0.02	–0.2	0.08	–0.43	–0.05	–0.24	0.13	0.12	–0.41	0.12	–0.05	0.00	–0.04	–0.36
*Enterobacter*	0.14	0.20	0.23	0.19	0.21	0.21	–0.27	0.00	–0.24	0.02	0.19	–0.04	0.5*	0.35	0.37	0.22	0.34
*Arthrobacter*	0.07	–0.01	0.02	0.07	–0.02	–0.04	−0.51*	0.02	–0.35	–0.1	0.14	–0.38	0.03	0.03	–0.04	0.01	–0.4
*Nitrosotalea*	−0.58*	−0.68*	–0.44	–0.3	–0.39	–0.38	–0.16	−0.59*	–0.11	–0.48	–0.42	–0.23	–0.39	−0.57*	–0.44	−0.59*	–0.31
*Massilia*	–0.23	0.11	0.28	0.10	0.13	0.27	−0.56*	0.21	–0.23	0.31	0.30	–0.34	0.23	0.14	0.14	0.15	–0.33
*Nitrobacter*	–0.47	−0.58*	–0.44	–0.31	−0.58*	−0.53*	–0.06	−0.57*	–0.32	–0.58	–0.41	–0.09	–0.4	−0.53*	−0.48*	−0.54*	–0.11
*Geobacter*	−0.51*	−0.62*	−0.59*	–0.42	−0.63*	−0.5*	–0.35	−0.58*	–0.32	–0.53	–0.39	–0.27	–0.38	−0.53*	−0.52*	−0.61*	–0.11
*Pseudomonas*	–0.14	–0.2	–0.38	–0.43	–0.13	–0.17	0.06	–0.19	–0.3	–0.11	–0.36	0.25	–0.35	–0.27	–0.2	–0.29	0.32

N, Nitrogen; P, Phosphorous; K, Potassium; TOC, Total organic carbon; SOC, Soil organic carbon; Fe, Iron; Ca, Calcium; Cu, Copper; DHA, Dehydrogenase activity; Mg, Magnesium; Mn, Manganese; S, sulfur; SMBC, Soil microbial biomass carbon; SMBN, Soil microbial biomass nitrogen; SMBP, Soil microbial biomass Phosphorous; Zn, Zinc. *Correlation significant at 0.05 level (two-tailed).

Moreover, significant negative correlations were also recorded between the relative abundance of Proteobacteria in rhizosphere and soil properties such as pH, N, P, K, Cu, and Mg, whereas the relative abundance of Thaumarchaeota in the rhizosphere was positively correlated with Mg and negatively correlated with Zn (Supplementary Table S4). The relative abundance of *Nitrosotalea* showed a significant positive correlation with Mn and a negative correlation with Zn in the rhizosphere. A significant negative correlation was recorded between the relative abundance of *Pseudomonas* and P, whereas a positive correlation was observed for *Bacillus* with Cu.

### Predictive Functional Analysis of the Microbial Community

The functional contributions of the bacteria were predicted based on OTUs, and the results revealed the presence of 398 different functional classes. Among these classes, transporters, two-component system, secretion system, ABC transporters, transcription factors, peptidases, ribosome, methane metabolism, quorum sensing, and bacterial motility proteins were the top 10 highly represented classes. The presence of 6596 KEGG orthologs was predicted across all samples, belonging to metabolism, environmental information processing, cellular processes, human diseases, genetic information processing, and organismal systems. PCA based on the highly abundant functions clustered the bulk soil and rhizosphere samples into separate groups (Supplementary Figure S3). The majority of the rhizosphere samples is placed on the negative side of PC1 and the positive side of PC2, whereas most of the bulk samples were on the positive side of PC1 and the negative side of PC2 (Supplementary Figure S3). Bacterial genes associated with iron complex outer membrane receptor protein, cobalt–zinc–cadmium resistance (CzcA) protein, RNA polymerase sigma-70 factor, ribonuclease E, translation initiation factor IF-2, serine/threonine-protein kinase, hydrophobic/amphiphilic exporter-1, beta-glucosidase, and multiple sugar transport system permease proteins showed high negative values for PC1 and positive for PC2 (Supplementary Table S5). Rhizosphere samples revealed high abundance of genes involved in nitrogen fixation (*NifQ*) by 64%, enterobactin (siderophore) production by 39%, plant hormone IAA production (tryptophan 2-monooxygenase) by 10%, and phosphate solubilization (pyrroloquinoline quinone C) by 09% in comparison to bulk soil samples (Supplementary Table S6).

## Discussion

Conservation agriculture offers a framework to improve soil structure, save water, enhance soil nutrient supply and cycling, increase yield, and maintain soil biodiversity. This study was designed to investigate the effect of long-term exposure to various tillage and residue management practices on the bacterial community structures of the bulk soils and how pea plant (a rotation crop) shapes the rhizosphere communities. Our results showed the dominance of Proteobacteria, Firmicutes, Acidobacteria, and Actinobacteria in both bulk and rhizosphere soils (Figure 3A) and are in agreement to the previous report on the dominance of copiotrophic microorganisms such as Proteobacteria, Firmicutes, and Actinobacteria in rich organic environments, and soils with low pH harbor more Acidobacteria (Fierer et al., 2007).

Significant differences were observed in the overall alpha diversity in the rhizosphere and bulk soil samples (Supplementary Figure S1), demonstrating higher species abundance and evenness (based on Shannon and Simpson indices) in rhizosphere samples. On the contrary, there was no significant difference in bacterial richness and evenness among the different tillage and residue management treatments (*P* > 0.05) in both rhizosphere and bulk soils (Supplementary Figure S1). This indicated that, in this study, the plant rhizosphere effect is the key driver for alpha diversity. Plants can alter the microbial communities by secreting a variety of nutrients and bioactive molecules into the rhizosphere (Hu et al., 2018; Huang et al., 2019). The enrichment of specific OTUs in the pea rhizosphere leading to the increased diversity was further confirmed, as all the rhizosphere samples were grouped in a small cluster, in comparison to a loose clustering of bulk soil samples in the PCoA biplot (Figure 1). The buildup of homogeneous bacterial communities in most of the rhizosphere samples can be attributed to the selection pressure of the pea roots, which continually release a large number of border cells and mucilage (Ropitaux et al., 2019), and pose a strong rhizosphere effect on the bacteria. In addition to this, the pea is a nitrogen-fixing crop, and an increase in the diversity of rhizosphere bacteria with soil mineral nitrogen levels has been observed in a study on frequency cropping of pulses (Hamel et al., 2018). Impact of crop plant on the diversity of rhizosphere microbes has also been observed in different crops including barley, cotton, maize, pulses, and wheat (Hamel et al., 2018; Yurgel et al., 2018; Babin et al., 2019; Kerdraon et al., 2019).

The majority of members of microbial communities in the host plant are horizontally acquired from the surrounding environment, and the soil is the main reservoir of a plant rhizosphere microbiome (Wagner et al., 2016; Sánchez-Cañizares et al., 2017). Our results on pea rhizosphere and bulk soils are consistent with this, as 551 (60%) genera of 917 were common in bulk and rhizosphere soil samples (Figure 3C). The dominance of Proteobacteria recorded in pea rhizosphere samples, with a significant increase in the abundance of genera including *Pseudomonas*, *Rhizobium*, *Pantoae*, *Enterobacter*, and *Sphingomonas* known for plant growth-promoting attributes, was in agreement with the previous studies on the rhizosphere microbiome analysis (Weinert et al., 2011; Mendes et al., 2013; Yurgel et al., 2018; Goss-Souza et al., 2019). Pea-*Rhizobium* symbiosis is well documented in Indian soils (Rahi et al., 2012); hence, an increase in the abundance of *Rhizobium* in the pea rhizosphere was expected. A higher abundance of *Pseudomonas* and *Sphingomonas* was also reported in the rhizospheres of crop plants such as lettuce, pea, wheat, and maize (Schreiter et al., 2014; Kerdraon et al., 2019). In addition to Proteobacteria, a significantly higher abundance of Bacteroidetes and Planctomycetes was also recorded in the rhizosphere soil in comparison to bulk soil samples (Figures 2, 3); positive correlations with increased abundance of Bacteroidetes and Planctomycetes have been observed to increase in organic carbon and phosphorus concentrations, respectively, in the rhizosphere of soybean (Goss-Souza et al., 2019). The genus *Nitrobacter* was at higher abundance in pea rhizosphere samples than bulk soils (Figure 3B), suggesting its enrichment by host plant as *Nitrobacter* converts nitrite to nitrate, making nitrogen more readily available to the host plant (Richardson et al., 2009; Hubbard et al., 2019).

Amplicon sequencing-based analysis revealed a higher abundance of phylum Firmicutes in the bulk soil samples in comparison to the rhizosphere samples, which was further substantiated with the similar results obtained by qPCR (Figure 4). The abundance of Firmicutes represented by members of genera, such as *Bacillus*, *Staphylococcus*, and *Planomicrobium*, proves a significant decrease in pea rhizosphere in comparison to bulk soil samples (Figures 2, 3); previous studies have also indicated a negative correlation of plant growth and high abundance of Firmicutes in the soil (Zhang et al., 2014; Kumar et al., 2018). The consistently higher relative proportion of genus *Planomicrobium* in the majority of bulk soil samples (Figure 2) also confirms its dominance in soils (Hui et al., 2019). The abundance of bacterial phyla Chloroflexi and Nitrospirae was significantly higher in bulk soil in comparison to rhizosphere samples (Figure 3A), which is in agreement with the previous study on the impact of land-use intensity and plant functional identity on microbial communities (Schöps et al., 2018). Both Chloroflexi and Nitrospirae are slow-growers adapted to low substrate concentrations (Daims et al., 2015) and could not cope up with other fast-growing bacterial communities in the nutrient-rich pea rhizosphere environment. Members of Nitrospirae are involved in nitrification process (oxidation of nitrite to nitrate) and have been reported to be dominant in N-fertilized treatment in paddy soil (Kumar et al., 2018). The significantly high abundance of *Massilia* and *Paenibacillus* was observed in bulk soil than rhizosphere samples (Figure 3B), which can be attributed to the presence of high cellulosic biomass in most of the treatment leading to the selection of the members of genera with potential to degrade cellulose (Ofek et al., 2012; Grady et al., 2016).

Minor differences were observed in the bacterial community composition in response to residue management treatments in both bulk and rhizosphere soils (Figure 5), exhibiting the complex responses of microbial communities to fertilizer applications (Hartmann et al., 2015). The higher proportion of *Pseudolabrys* in 100% NPK and organic treatments indicate its specific enrichment in these treatments to higher levels of nitrogen (Figure 5); previous studies based on the increased abundance of genera *Pseudolabrys*, *Elstera*, and *Ramlibacter* suggested them to be used as bacterial biomarkers for different nitrogen levels (Yan et al., 2017). *Roseiarcus* was abundant in the organic treatment (Figure 5). The member of this genus was isolated from acidic peat soil (Kulichevskaya et al., 2014). The function of this genus is not well understood. Higher abundance of *Tumebacillus* was recorded in organic treatment (Figure 5). *Tumebacillus* is a highly abundant member of Firmicutes in different soils (Lian et al., 2019). Among the rhizosphere sample, the significantly higher relative abundance of *Anaerolineae*, *Hydrogenispora*, and *Syntrophorhabdus* was observed for the organic treatment (Figure 5), all of these genera are capable of decomposing diverse carbon sources under anoxic environments and have been identified as the unique core taxa for rice soils (Jiao et al., 2019).

Although pea was cultivated under zero tillage, the influence of previous tillage treatments during rice cultivation was observed on the prevalence of few specific OTUs (Supplementary Figure S2), suggesting the plausible effect of tillage treatments on the bacterial community structure. This can be attributed to the fact that tillage practices alter soil bulk density, pore structure, water availability, soil organic carbon, and so on (Zhang et al., 2018). However, the impact to tillage treatments was not pronounced, and only 0.56% OTUs in the bulk soil and 2.60% OTUs in the rhizosphere soil were enriched across various tillage treatments (Supplementary Figure S2). Enrichment of OTUs assigned to different genera was recorded in the rhizosphere soil samples in the CT in comparison to ZT and MT (Table 2), which can be attributed to the fact that intense soil disturbance in conventional tillage accelerates soil organic matter oxidation, hence expected to enrich diverse member of bacteria (Ding et al., 2011).

The levels of N, P, K, and few micronutrients showed a significant negative correlation with the relative abundance of Proteobacteria in pea rhizosphere (Supplementary Table S4); a strong negative correlation was recorded between the relative abundance of Proteobacteria and available phosphorus in *Pinus tabuliformis* forest soils (Deng et al., 2018). Among the members of Proteobacteria, with the relative abundance of genus *Pseudomonas*, a significant negative correlation was recorded with values of P (Supplementary Table S4), exhibiting the enrichment of phosphate-solubilizing *Pseudomonas* in the pea rhizosphere in the soils with lower *P*-values (Gulati et al., 2008). The results of our study showed a significant negative correlation of multiple soil nutrients values with the relative abundance of Chloroflexi, and the level of N with Acidobacteria in the bulk soil (Table 3) is in agreement to the reports that Chloroflexi and Acidobacteria are generally oligotrophic and had slower growth rates (Fierer et al., 2012). Planctomycetes in soils has been reported to be sensitive to soil history (Buckley and Schmidt, 2003); in our study, we found a significant negative correlation between the abundance of Planctomycetes in bulk soil and multiple parameters of soil nutrients (Table 3). Members of Chloroflexi and Planctomycetes have been reported for negative responses to fertilization (Eo and Park, 2016). Similar negative correlation to various soil properties, including available N, has been observed for *Nitrobacter* and *Nitrosotalea* (Table 3), as the members of these genera are known as nitrite and ammonium oxidizers, and contributes to nitrification in soils (Pajares and Bohannan, 2016).

Higher abundance of genes related to nitrogen fixation, phytohormone and siderophore production, and phosphate solubilization in the rhizosphere soil (Supplementary Table S6) substantiate our earlier conclusions on the selection of bacterial communities with plant growth-promoting potential in the rhizosphere (Figure 3). The selection of specific bacterial communities in the rhizosphere based on their putative functions was further confirmed by specific clustering of the majority of rhizosphere and a bulk sample in the PCA biplot constructed based on the highly abundant functions (Supplementary Figure S3). In addition to the high abundance of plant growth promotion genes, bacterial genes associated with iron complex outer membrane receptor protein, CzcA; RNA polymerase sigma-70 factor; and ribonuclease E were also abundant in rhizosphere soil as compared to the bulk soil (Supplementary File S1), revealing the possible role of bacterial communities in abiotic stress (low pH and high aluminum and iron toxicity) amelioration. The higher abundance of iron complex outer membrane receptor protein in the rhizosphere indicates the enrichment of Gram-negative bacteria, which outer membrane receptor system for siderophore-mediated Fe transportation (Ahmed and Holmström, 2014). The CzcA protein has been reported to be involved in the efflux of heavy metals ions (Nies, 2000) and was also present in metal resistance *Pseudomonas putida* S13.1.2 isolated from a vineyard soil (Chong et al., 2016). Rhizosphere offers a variety of abiotic stress, including temperature fluctuations, salinity, osmolarity, oxygen concentration, and nutrient concentration, to the bacteria; RNA polymerase sigma-70 factors play relevant roles in adapting to different kinds of stresses (Martínez-Salazar et al., 2009; Mishra et al., 2011).

## Conclusion

Our work showed that pea plant is the most dominating selection factor shaping the microbial communities under diverse residue management and tillage treatments. Soil management and residue management practices also affect the bacterial community structure in both bulk soil and pea rhizosphere. Enrichment of bacterial taxa known for plant growth promotion attributes and removal of toxic elements from soil was recorded in the rhizosphere, indicating selection of rhizosphere communities by the plant to meet its requirements of nutrient uptake and combating stress. Predictive functional analysis also revealed the plausible enrichment of plant growth-promoting and stress tolerance genes in the pea rhizosphere.

## Data Availability Statement

The datasets generated for this study can be found in the sequence data is made available at NCBI SRA submission with accession number SUB5624752 (Bioproject ID: PRJNA544901).

## Author Contributions

PR, KR, AD, YS, and JL conceived the study. KR, SB, and BK provided the samples. KR, AD, JL, SB, and BK performed the soil physio-chemical analysis. DC performed the bioinformatics and statistical analyses. PR participated and provided guidance with the data analysis. PR and DC drafted the manuscript. KR contributed to manuscript revisions. All authors have read and approved the final version of the manuscript.

## Conflict of Interest

The authors declare that the research was conducted in the absence of any commercial or financial relationships that could be construed as a potential conflict of interest.

## Footnotes

1 http://www.interactivenn.net
