# Supplementary material for: Pea (Pisum sativum l.) Plant Shapes Its Rhizosphere Microbiome for Nutrient Uptake and Stress Amelioration in Acidic Soils of the North-East Region of India
Source: Front Microbiol. 2020 Jun 3;11:968. doi: 10.3389/fmicb.2020.00968 (PMC7283456; doi:10.3389/fmicb.2020.00968)
Supplement: Supplementary file 1 [file Data_Sheet_1.PDF]

Supplementary information

Pea (*Pisum sativum* l.) Plant Shapes its Rhizosphere Microbiome for Nutrient Uptake and Stress Amelioration in Acidic Soils of the North-East Region of India

Diptaraj Chaudhari[1], Krishnappa Rangappa[2], Anup Das[2], Jayanta Layek[2], Savita Basavaraju[2], Basant Kumar Kandpal[2], Yogesh Shouche[1], Praveen Rahi[1]*

[1]National Center for Microbial Resource, National Center for Cell Science, Pune

[2]ICAR Research Complex for NER Region, Umiam, 793 103, Meghalaya, India

***Corresponding Author:**

Dr. Praveen Rahi

National Center for Microbial Resource,

National Center for Cell Science, Pune, India

*Email ID: praveen_rahi22@yahoo.co.in; praveen@nccs.res.in

Table S1: qPCR primers used in this study.

Bacteria taxa	Primer	Primer sequence (5'→3')	Amplicon size	Annealing temperature
Bacteria	Bact341F	CCTACGGGAGGCAGCAG	177	60
	Bact518R	ATTACCGCGGCTGCTGG		
Firmicutes	Firm934F	GGAGYATGTGGTTTAATTCGAAGCA	126	60
	Firm1060R	AGCTGACGACAACCATGCAC		

Detailed chemical properties of soil:

Exchangeable Ca and Mg values were recorded higher under NT compared to MT and CT. Among the nutrient management practices, exchangeable Ca at 0-15 cm of the soil was higher under 50 % NPK + WB (2.36 cmol (P^+)/kg of soil) which followed by 50 % NPK + GLM (2.33 cmol (P^+)/kg of soil). Significantly higher exchangeable Mg recorded under 50%NPK+WB (2.18 cmol (P^+)/kg of soil) at depth 0-15 cm was at par with 50% NPK+ FYM+WB+RP (2.14 cmol (P^+)/kg of soil) and 50% NPK+ GLM (2.12 cmol (P^+)/kg of soil) compared to 100% NPK under NT practices. Available sulphur at 0-15 cm of the soil was higher under MT + 50 % NPK + FYM+WB+RP (7.03 mg/kg) followed by NT for 50 % NPK + FYM+WB+RP (6.98 mg/kg). The interaction effect of tillage and nutrient management practices on Exchangeable Ca, Mg and S were observed significant (Table S2). Soil nutrient estimates revealed that Fe, Mn, and Zn at 0-15cm were significantly influenced by tillage and nutrient management practices (Table S2). The interaction effect of tillage and nutrient management practices on Fe, Mn, and Zn were significant. The higher available Fe and Mn at 0-15 cm depth of soil were recorded under 100 % NPK with NT practices and 100 % NPK + GLM with MT practices respectively.

In the similar line the values of Cu, SMBC, SMBN, SMBP and DHA at 0-15cm were significantly influenced by tillage and nutrient management practices (Table S3). Among the nutrient management practices, available Cu at 0-15 cm of the soil was the higher under 50 % NPK + GLM (7.63 mg/kg) which is statistically on par with 50 % NPK + FYM+WB + RP (7.52 mg/kg) and 50 % NPK + WB (7.51 mg/kg). The interaction effect of tillage and nutrient management practices on SMBC was recorded significant. Among the nutrient management practices, SMBC at 0-15 cm of the soil was the higher under MT for 50 % NPK + FYM+WB + RP (183.0 µg/g dry soil) followed by NT + 50 % NPK + GLM (176.5 µg/g

dry soil) (Table S3). The lowest SMBC at 0-15 cm was recorded under CT + 100 % NPK 128.4 μg/g dry soil.

Table S2: Nutrient content of soils collected from different treatment plots.

Tillage	Ca (cmole(p+)/kg soil)				Mg (cmole(p+)/kg soil)				S (mg/kg soil)			
**Nutrient Inputs**	**ZT**	**MT**	**CT**	**Mean**	**ZT**	**MT**	**CT**	**Mean**	**ZT**	**MT**	**CT**	**Mean**
100% NPK	2.09	2.01	1.97	2.02	1.87	1.83	1.83	1.84	5.90	5.72	5.67	5.76
50% NPK	2.01	1.95	1.91	1.96	1.86	1.77	1.78	1.80	5.84	5.58	5.34	5.59
50% NPK+ISRR	2.27	2.17	2.18	2.21	2.07	1.98	1.86	1.97	6.33	6.25	5.81	6.13
50% NPK+WB	2.36	2.20	2.17	2.24	2.12	2.05	1.90	2.03	6.45	6.33	6.01	6.26
50% NPK+GLM	2.33	2.27	2.25	2.28	2.18	2.02	1.91	2.04	6.79	6.44	6.12	6.45
FYM+WB+RP	2.21	2.11	2.10	2.14	2.14	1.99	1.86	1.99	6.98	7.03	6.32	6.78
**Mean**	**2.21**	**2.12**	**2.10**		**2.04**	**1.94**	**1.86**		**6.38**	**6.23**	**5.88**	
**Variant**	**S.Em±**		**C.D.** (p=0.05)		**S.Em±**		**C.D.** (p=0.05)		**S.Em±**		**C.D.** (p=0.05)	
Tillage	0.03		0.09		0.03		0.10		0.10		0.30	
Nutrient Inputs	0.04		0.06		0.05		0.07		0.15		0.21	
Interactions	0.08		0.22		0.08		0.24		0.25		0.73	
	**Fe (mg/kg soil)**				**Mn (mg/kg soil)**				**Zn (mg/kg soil)**			
	**ZT**	**MT**	**CT**	**Mean**	**ZT**	**MT**	**CT**	**Mean**	**ZT**	**MT**	**CT**	**Mean**
100% NPK	123.2	158.4	126.4	136.0	5.32	5.73	5.24	5.43	1.07	1.34	0.77	1.06
50% NPK	98.5	132.3	119.3	116.7	3.73	5.49	4.81	4.68	0.39	0.64	0.79	0.61
50% NPK+ISRR	154.3	113.3	127.9	131.9	4.31	4.33	5.90	4.85	0.78	0.51	0.75	0.68
50% NPK+WB	144.4	142.2	180.8	155.8	4.58	6.84	8.46	6.62	0.72	1.44	1.47	1.21
50% NPK+GLM	105.8	113.9	140.1	119.9	4.44	7.15	7.46	6.35	0.69	0.79	1.67	1.05
FYM+WB+RP	174.0	125.7	128.7	142.8	5.22	5.71	5.86	5.60	0.79	1.61	1.26	1.22
**Mean**	**133.4**	**131.0**	**137.2**		**4.60**	**5.88**	**6.29**		**0.74**	**1.06**	**1.12**	
**Variant**	**S.Em±**		**C.D.** (p=0.05)		**S.Em±**		**C.D.** (p=0.05)		**S.Em±**		**C.D.** (p=0.05)	
Tillage	5.2		14.9		0.25		0.73		0.07		0.21	
Nutrient Inputs	7.3		10.4		0.36		0.51		0.1		0.14	
Interactions	12.7		36.6		0.62		1.78		0.18		0.51	

*Abbreviation: Ca-Calcium, Mg-Magnesium, S-Sulphur, Fe-Iron, Mn-Manganese, Zn-Zinc, NPK-Nitrogen, Phosphorus, K-Potassium, ISRR-*In-situ* Rice Residue Retention, WB-Weed Biomass, GLM-Green Leaf Manure, RP-Rock Phosphate, ZT-Zero Tillage, MT-Minimum Tillage, CT-Conventional tillage

Table S3: Copper content and biological properties of soils collected from different treatment plots.

Tillage	Cu (mg/kg soil)				SMBC (µg/g dry soil)				SMBN (µg/g dry soil)			
**Nutrient Inputs**	**ZT**	**MT**	**CT**	**Mean**	**ZT**	**MT**	**CT**	**Mean**	**ZT**	**MT**	**CT**	**Mean**
100% NPK	1.45	1.38	1.21	1.35	155.3	140.3	141.4	145.6	58.4	56.0	54.3	56.2
50% NPK	1.10	1.33	1.28	1.23	139.2	130.0	128.4	132.5	57.3	55.8	48.3	53.8
50% NPK+ISRR	1.66	1.19	1.28	1.38	171.3	166.7	166.7	168.2	62.9	63.0	61.3	62.4
50% NPK+WB	1.55	1.31	1.40	1.42	167.3	162.7	159.1	163.0	65.3	63.6	62.1	63.7
50% NPK+GLM	1.36	1.30	1.30	1.32	176.5	172.1	172.2	173.6	67.3	63.7	64.8	65.3
FYM+WB+RP	1.64	1.36	1.40	1.46	174.0	183.0	170.7	175.9	69.6	68.0	61.3	66.3
**Mean**	1.46	1.31	1.31		163.9	159.1	156.4		63.4	61.7	58.7	
**Variant**	**S.Em±**		**C.D.** (p=0.05)		**S.Em±**		**C.D.** (p=0.05)		**S.Em±**		**C.D.** (p=0.05)	
Tillage	0.03		0.10		1.2		3.4		0.6		1.7	
Nutrient Inputs	0.05		0.07		1.7		2.4		0.8		1.2	
Interactions	0.08		0.24		2.9		8.3		1.5		4.2	
	**SMBP (µg/g dry soil)**				**DHA (µg/g dry soil/hr)**							
	**ZT**	**MT**	**CT**	**Mean**	**ZT**	**MT**	**CT**	**Mean**				
100% NPK	6.39	5.87	5.77	6.01	22.3	18.4	17.0	19.3				
50% NPK	5.97	5.47	5.24	5.56	21.0	18.0	17.0	18.7				
50% NPK+ISRR	7.28	7.05	6.86	7.07	25.2	19.0	23.0	22.4				
50% NPK+WB	7.51	7.17	7.18	7.29	26.9	21.1	22.7	23.6				
50% NPK+GLM	7.63	7.16	7.09	7.29	28.4	21.9	25.0	25.1				
FYM+WB+RP	7.52	7.17	6.79	7.16	25.3	22.2	20.3	22.6				
**Mean**	7.05	6.65	6.49		24.9	20.1	20.8					
**Variant**	**S.Em±**		**C.D.** (p=0.05)		**S.Em±**		**C.D.** (p=0.05)					
Tillage	0.07		0.21		0.6		1.8					
Nutrient Inputs	0.11		0.15		0.9		1.2					
Interactions	0.18		0.52		1.5		4.4					

*Abbreviation: Cu-Copper, SMBC-Soil Microbial Biomass Carbon, SMBN-Soil Microbial Biomass Nitrogen, SMBP-Soil Microbial Biomass Phosphorus, DHA-Dehydrogenase Activity, NPK-Nitrogen, Phosphorus, K-Potassium, ISRR-*In-situ* Rice Residue Retention, WB-Weed Biomass, GLM-Green Leaf Manure, RP-Rock Phosphate, ZT-Zero Tillage, MT-Minimum Tillage, CT-Conventional tillage.

Table S4: Pearson's correlation coefficient (R) between the relative abundance of rhizosphere bacterial taxa and measured soil characteristics.

**Bacterial taxa**	**Measured soil characteristics**																
	**pH**	**N**	**P**	**K**	**TOC**	**SOC**	**Fe**	**Ca**	**Cu**	**DHA**	**Mg**	**Mn**	**S**	**SMBC**	**SMBN**	**SMBP**	**Zn**
**Phylum**																	
Proteobacteria	**-0.49***	**-0.47***	**-0.57***	**-0.53***	-0.29	-0.35	-0.29	-0.39	**-0.52***	-0.34	**-0.5***	0.13	-0.32	-0.3	-0.24	-0.37	-0.16
Firmicutes	0.46	0.37	0.39	0.43	0.24	0.13	0.35	0.25	0.42	0.13	0.31	-0.07	0.08	0.09	0.08	0.19	0.14
Acidobacteria	-0.08	-0.07	-0.02	-0.05	-0.14	0.17	-0.04	0.01	0.01	0.15	0.07	0.01	0.32	0.25	0.21	0.14	0.09
Actinobacteria	-0.39	-0.08	-0.11	-0.27	-0.07	0.13	-0.39	0.01	-0.28	-0.01	-0.12	0.19	-0.05	0.05	-0.09	-0.04	-0.14
Chloroflexi	-0.05	0.00	0.14	0.13	0.07	0.33	0.17	0.01	0.36	0.22	0.20	-0.08	0.42	0.24	0.28	0.15	0.23
Nitrospirae	0.05	-0.1	-0.27	-0.21	-0.23	0.00	0.06	-0.12	-0.17	-0.12	-0.18	0.32	0.13	0.20	0.09	0.03	0.38
Verrucomicrobia	-0.07	-0.04	0.04	-0.07	-0.1	0.17	-0.17	0.09	0.00	0.30	0.12	-0.09	0.28	0.23	0.20	0.16	-0.01
Thaumarchaeota	0.00	0.32	0.42	0.30	0.40	0.44	-0.46	0.46	-0.15	0.39	**0.5***	-0.3	0.37	0.29	0.36	0.37	**-0.51***
Bacteroidetes	-0.23	-0.25	-0.22	-0.43	-0.25	-0.19	-0.41	-0.17	-0.39	0.08	-0.22	-0.07	-0.14	-0.07	-0.17	-0.14	-0.03
Planctomycetes	-0.02	-0.1	0.06	0.03	-0.08	0.20	0.06	-0.04	0.16	0.14	0.08	-0.11	0.32	0.20	0.21	0.11	0.08
**Genera**																	
Bacillus	0.44	0.26	0.36	0.41	0.24	0.20	0.36	0.22	**0.57***	0.20	0.22	-0.26	0.03	0.07	0.02	0.12	-0.04
Staphylococcus	0.15	0.26	0.16	0.15	0.09	-0.08	0.09	0.11	-0.09	-0.06	0.21	0.25	0.09	0.04	0.11	0.15	0.30
Planomicrobium	-0.21	0.04	-0.08	-0.21	-0.16	0.05	-0.18	0.14	0.03	0.19	-0.01	0.11	-0.05	0.11	-0.08	0.05	0.01
Enterobacter	-0.3	-0.32	-0.42	-0.3	-0.09	-0.3	0.03	-0.32	-0.19	-0.36	-0.36	0.10	-0.32	-0.39	-0.21	-0.35	-0.1
Arthrobacter	-0.32	0.08	0.03	-0.12	0.18	0.31	-0.33	0.11	-0.16	0.01	0.01	0.24	0.04	0.08	0.01	0.03	-0.11
Nitrosotalea	0.02	0.33	0.42	0.31	0.41	0.44	-0.45	0.47	-0.14	0.39	**0.5***	-0.3	0.37	0.30	0.37	0.38	**-0.5***
Massilia	-0.3	0.03	-0.11	-0.2	-0.01	0.19	-0.31	0.09	-0.15	-0.03	-0.02	0.26	-0.05	0.03	-0.09	-0.03	-0.14
Nitrobacter	-0.11	-0.1	-0.14	-0.16	-0.35	0.07	-0.02	0.00	0.00	0.10	-0.04	0.17	0.16	0.21	0.08	0.09	0.12
Geobacter	0.10	-0.1	-0.28	-0.25	-0.27	-0.09	-0.02	-0.11	-0.21	-0.05	-0.13	0.18	0.11	0.17	0.05	0.03	0.25
Pseudomonas	-0.43	-0.36	**-0.47***	-0.39	-0.09	-0.26	0.08	-0.35	-0.14	-0.32	-0.43	0.16	-0.36	-0.43	-0.25	-0.41	-0.02

N, Nitrogen; P, Phosphorous; K, Potassium; TOC, Total organic carbon; SOC, Soil organic carbon; Fe, Iron; Ca, calcium; Cu, Copper; DHA, Dehydrogenase activity; Mg, Magnesium; Mn, manganese; S, sulfur; SMBC, Soil microbial biomass carbon; SMBN, Soil microbial biomass nitrogen; SMBP, Soil microbial biomass Phosphorous; Zn, Zinc. *Correlation significant at 0.05 level (two-tailed)

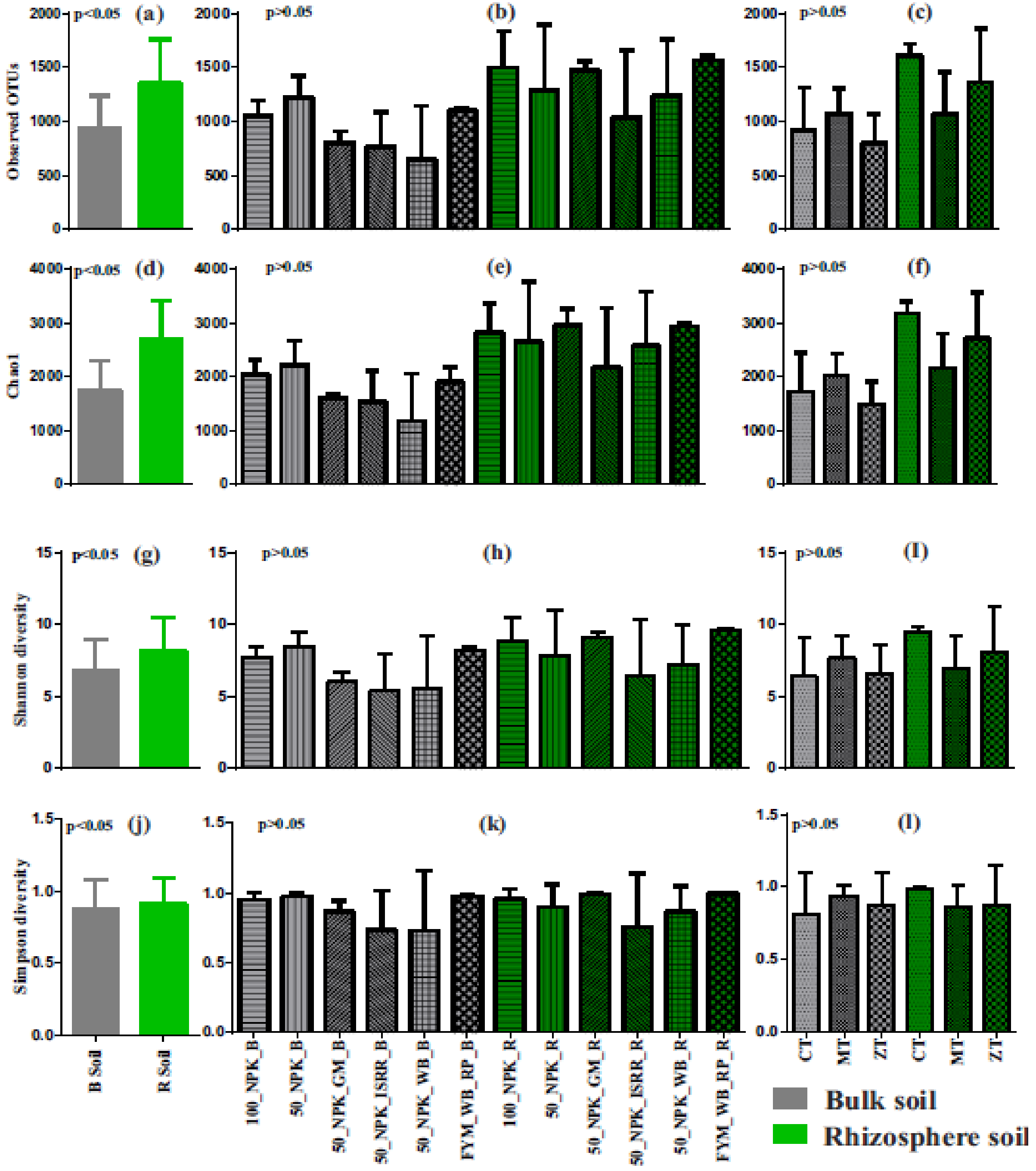

Fig. S1 Bar charts with standard error bars illustrating the values of alpha diversity measures, Observed OTUs (a-c) Chao1 (d-f), Shannon (g-i) and Simpson (j-l) in the bulk soil and pea rhizosphere samples from different tillage and residue management practices.

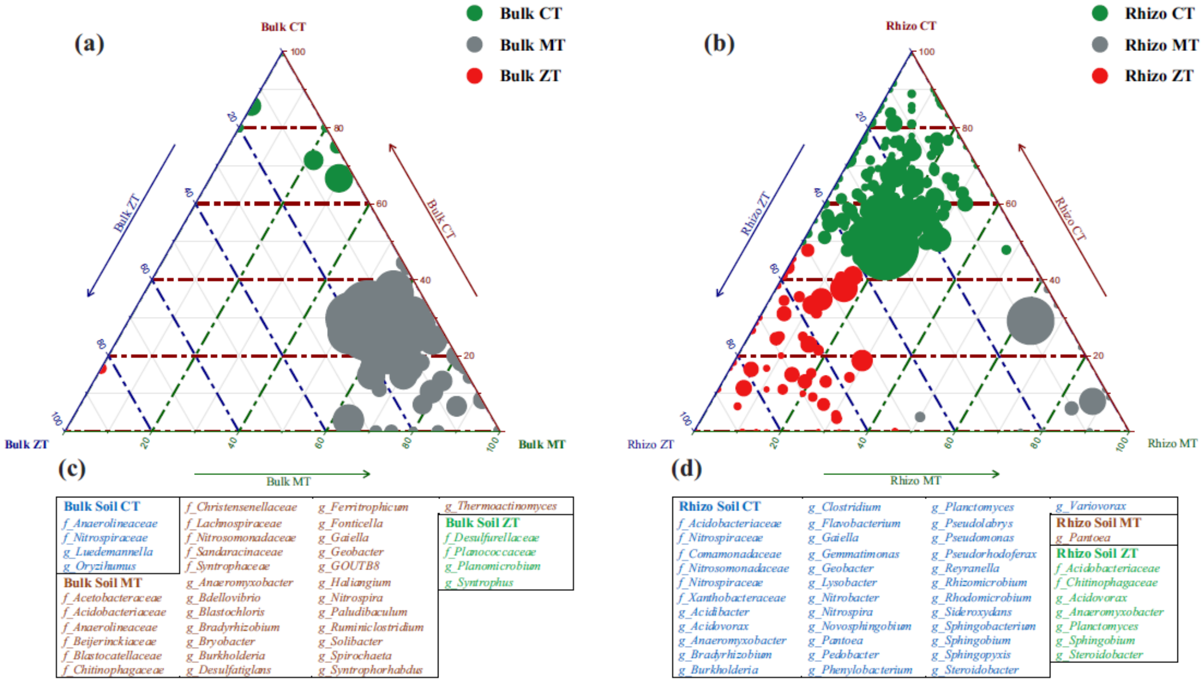

Fig. S2 Ternary plot depicting the number of OTUs enriched in the no-till, minimum tillage and conventional tillage plots in the a) bulk soil and b) pea rhizosphere samples. Each circle depicts one individual OTU. The size of the circle reflects its relative abundance. Family or genus level taxonomy of the OTUs specifically enriched in the bulk soil (c) and rhizosphere soil (d) in respective tillage treatments. For the rhizosphere soil samples taxonomy of OTUs having abundance more than 0.05% is represented.

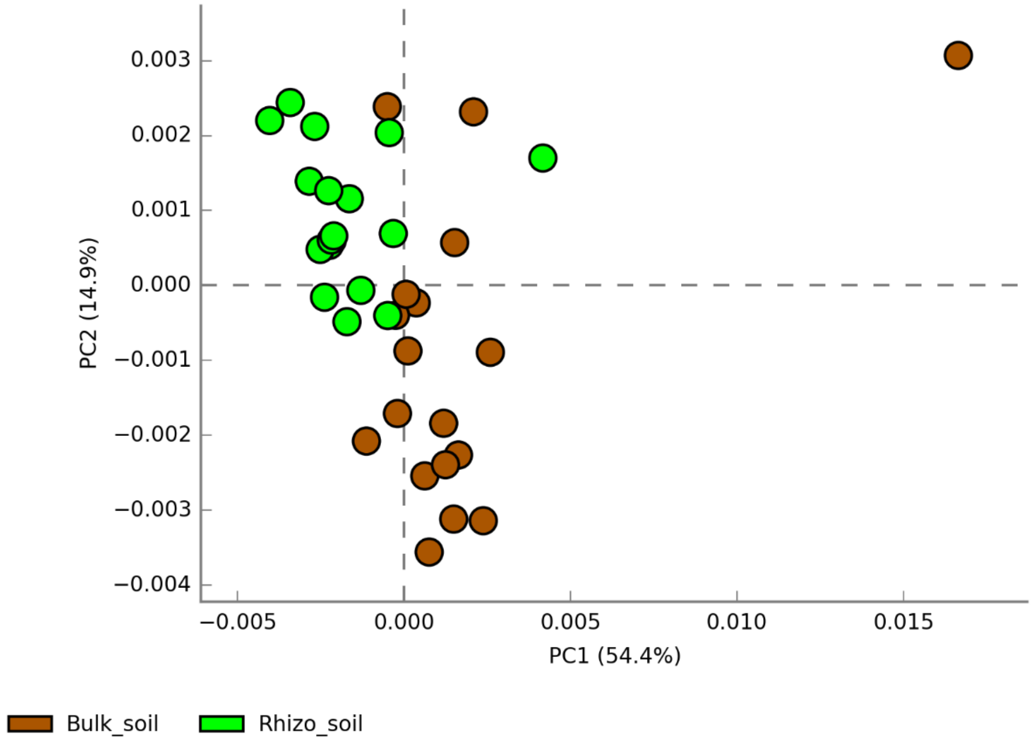

Fig. S3 PCA bi-plot based on relative abundance predictive metabolic potentials of bacterial communities in the bulk soil and pea rhizosphere samples.

Table S5. Loading values of the first two components of principalcomponent analysis (PCA) of predictive metabolic potentials of bacterial communities in the bulk soil and pea rhizosphere samples,ranked by PC1 loading.

**Functional Trait**	**PC 1**	**PC 2**
3-oxoacyl-[acyl-carrier protein] reductase	-0.24	0.08
cobalt-zinc-cadmium resistance protein CzcA	-0.24	0.05
RNA polymerase sigma-70 factor, ECF subfamily	-0.23	0.14
iron complex outermembranerecepter protein	-0.22	-0.09
ribonuclease E	-0.22	0.06
putative transposase	-0.18	-0.23
translation initiation factor IF-2	-0.16	0.14
serine/threonine protein kinase, bacterial	-0.15	0.25
hydrophobic/amphiphilic exporter-1	-0.15	0.11
beta-glucosidase	-0.14	0.25
branched-chain amino acid transport system substrate-binding protein	-0.14	-0.17
multiple sugar transport system permease protein	-0.12	0.14
putative ABC transport system permease protein	-0.06	0.26
branched-chain amino acid transport system permease protein	-0.04	-0.21
branched-chain amino acid transport system ATP-binding protein	-0.03	-0.17
peptide/nickel transport system substrate-binding protein	-0.03	-0.15
excinuclease ABC subunit A	0.04	0.33
Cu2+-exporting ATPase	0.05	-0.06
putative ABC transport system ATP-binding protein	0.07	0.27
peptide/nickel transport system permease protein	0.09	-0.19
formate dehydrogenase, alpha subunit	0.10	0.24
carbamoyl-phosphate synthase large subunit	0.11	0.27
ribonucleoside-diphosphatereductase alpha chain	0.11	0.23
ATP-dependent Lon protease	0.12	0.26
peptide/nickel transport system ATP-binding protein	0.13	-0.12
methyl-accepting chemotaxis protein	0.15	0.06
ATP-binding cassette, subfamily B, bacterial	0.17	-0.15
DNA helicase II / ATP-dependent DNA helicase PcrA	0.22	0.11
GTP-binding protein	0.24	-0.10
elongation factor G	0.25	0.08
DNA-directed RNA polymerase subunit beta	0.25	0.01
DNA polymerase III subunit alpha	0.26	-0.02
DNA-directed RNA polymerase subunit beta'	0.26	-0.01
phosphoribosylformylglycinamidine synthase	0.26	0.04

Table S6. Higher abundance of predicted genes with plant growth promoting functions in rhizosphere in comparison to bulk soil.

Treatment No	MFS transporter, ENTS family, enterobactin (siderophore)		Tryptophan 2-monooxygenase (IAA production)		Nitrogen fixation protein NifQ (N-fixation)		Pyrroloquinoline quinone C (P solubilization)	
	**Bulk Soil**	**Rhizo Soil**	**Bulk Soil**	**Rhizo Soil**	**Bulk Soil**	**Rhizo Soil**	**Bulk Soil**	**Rhizo Soil**
**1**	1.99E-05	2.61E-05	9.04E-06	1.35E-05	7.6E-06	1.9E-05	9.4E-05	8.8E-05
**2**	2.69E-05	2.12E-05	1.34E-05	1.26E-05	1.2E-05	2.0E-05	9.4E-05	1.0E-04
**3**	1.77E-06	2.18E-05	1.01E-06	9.22E-06	1.6E-06	1.6E-05	9.2E-06	9.3E-05
**4**	4.36E-05	3.27E-05	2.21E-05	1.51E-05	1.3E-05	2.1E-05	1.2E-04	9.7E-05
**5**	1.34E-05	2.70E-05	7.27E-06	1.06E-05	9.1E-06	1.5E-05	8.1E-05	9.0E-05
**6**	2.01E-05	1.35E-05	1.27E-05	9.89E-06	1.1E-05	1.8E-05	9.5E-05	8.9E-05
**7**	2.74E-05	3.15E-05	1.15E-05	1.07E-05	1.4E-05	1.5E-05	8.6E-05	8.7E-05
**8**	1.92E-05	7.93E-05	9.62E-06	1.11E-05	1.2E-05	1.5E-05	8.4E-05	7.6E-05
**9**	1.59E-05	2.36E-05	7.76E-06	5.81E-06	1.1E-05	9.9E-06	8.1E-05	4.9E-05
**10**	2.82E-05	1.73E-05	1.61E-05	1.28E-05	1.1E-05	2.2E-05	8.7E-05	1.1E-04
**11**	2.08E-05	3.28E-05	6.58E-06	7.92E-06	1.1E-05	1.5E-05	8.3E-05	7.2E-05
**12**	2.57E-05	2.97E-05	6.92E-06	8.01E-06	8.4E-06	1.6E-05	7.5E-05	8.5E-05
**13**	2.84E-05	3.58E-05	9.78E-06	1.21E-05	1.3E-05	1.7E-05	9.0E-05	8.7E-05
**14**	2.53E-05	3.40E-05	6.63E-06	1.02E-05	1.1E-05	2.0E-05	8.3E-05	9.2E-05
**15**	1.27E-05	3.84E-05	1.02E-05	1.11E-05	9.5E-06	1.8E-05	5.4E-05	9.2E-05
**16**	2.78E-05	4.10E-05	5.82E-06	9.21E-06	1.0E-05	1.9E-05	7.8E-05	9.4E-05
**17**	2.33E-05	3.00E-05	1.12E-05	1.09E-05	1.3E-05	1.8E-05	9.4E-05	9.3E-05
**18**	2.85E-05	3.16E-05	8.99E-06	1.32E-05	1.2E-05	2.0E-05	7.2E-05	9.3E-05
**Mean**	2.27E-05	3.15E-05	9.81E-06	1.08E-05	1.1E-05	1.7E-05	8.1E-05	8.8E-05
**% higher abundance**		**38.7**		**9.8**		**64.3**		**8.7**
